# Supplementary material for: All-optical control of lead halide perovskite microlasers
Source: Nat Commun. 2019 Apr 16;10:1770. doi: 10.1038/s41467-019-09876-6 (PMC6467983; doi:10.1038/s41467-019-09876-6)
Supplement: Supplementary file 1 — Supporting Information [file 41467_2019_9876_MOESM1_ESM.pdf]

Supplementary Information

All-optical control of lead halide perovskite microlasers

Zhang et al.

Supplementary Figures

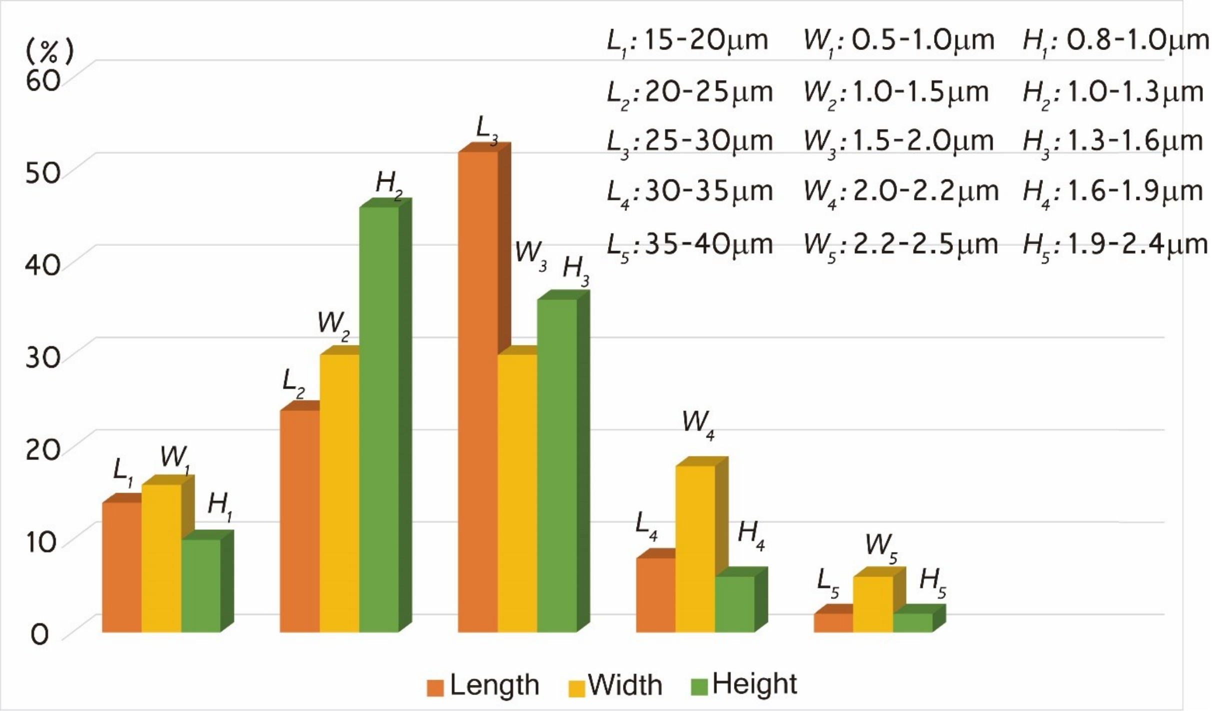

Supplementary Figure 1. Statistic graph showing width, length and thickness of synthesized microplates.

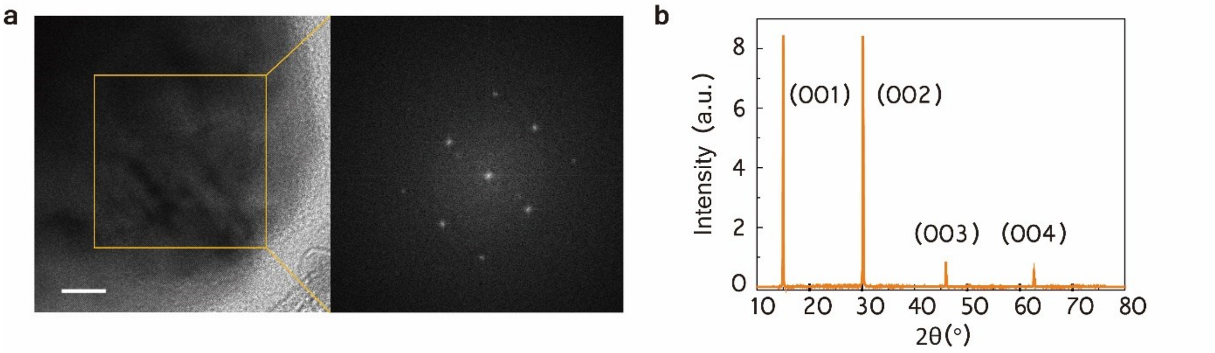

Supplementary Figure 2. The synthesized $MAPbBr_3$ perovskite microrods. (a) The high-resolution transimission electron microscopy (HRTEM) image and corresponding fast Fourier transform (FFT) pattern of $MAPbBr_3$ perovskite. The scale bar is 10 nm. (b) The X-ray diffraction (XRD) spectrum of synthesized perovskite microrods. Four sharp peaks appear at 15°, 30°, 46°, and 63°, which can be indexed to the (001), (002), (003), and (004) crystal planes of cubic phase of $MAPbBr_3$ perovskites.

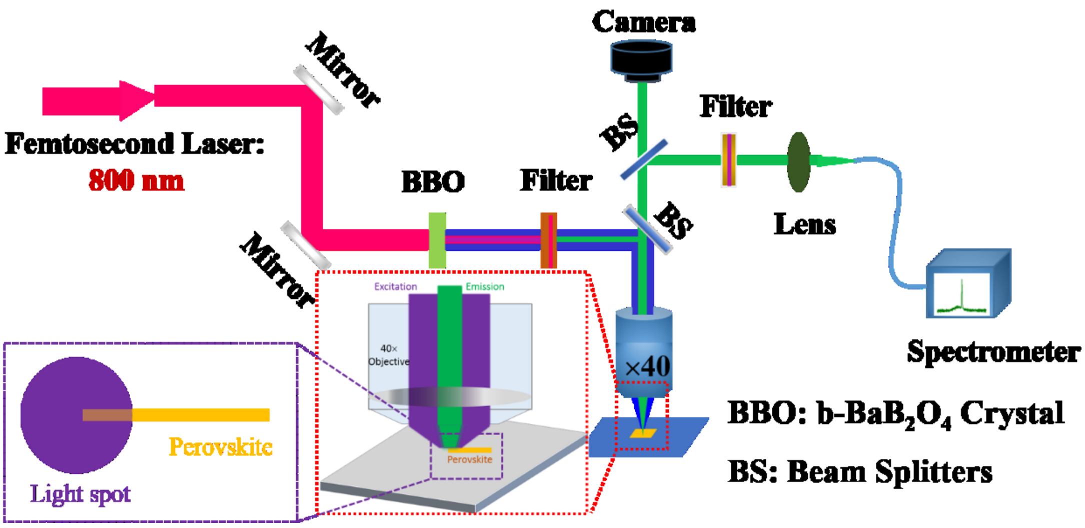

Supplementary Figure 3. Schematic diagram of the experimental setup for optically pumped fluorescence and lasing measurement. The inset is an enlarged image of the objective and the position of the pump light spot and perovskite microwire. The magnification of the objective is 40. The purple area represents excitation light and the green area represents the emission light.

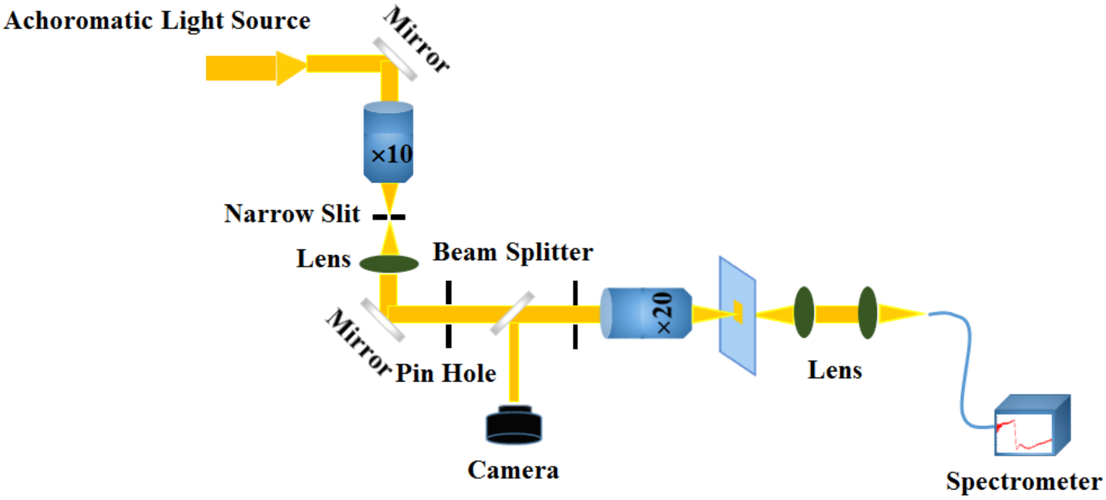

Supplementary Figure 4. Optical setup for measuring the absorption spectrum of a single microplate and microwire.

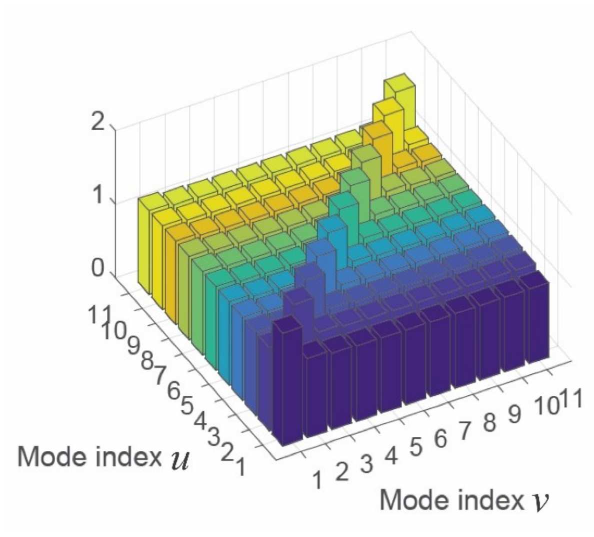

Supplementary Figure 5. Self- and cross-interaction coefficients for the first 11 modes in a Fabry-Perot cavity.

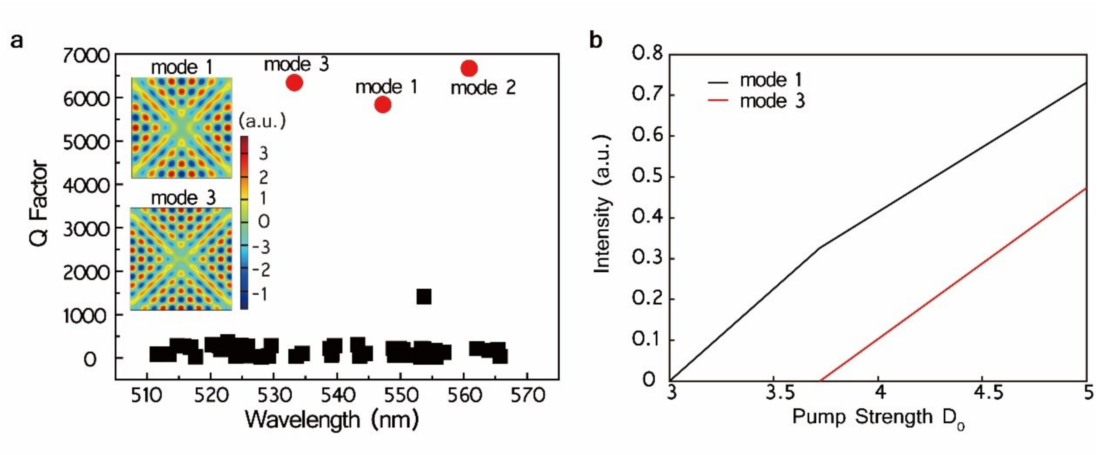

Supplementary Figure 6. Numerically analysis of the other adjacent high Q modes. (a) The numerically calculated Q factors in the cross-section of $MAPbBr_3$ perovskite microrod. (b) The output intensity of mode 1 and 3 as a function of pumping power when the modal interaction is considered.

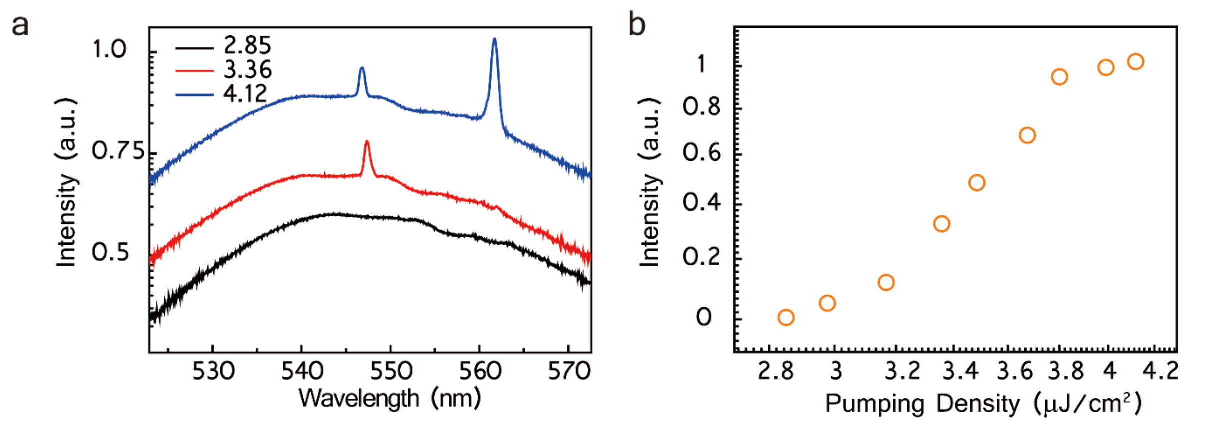

Supplementary Figure 7. The optically characteristics perovskite microlasers. (a) The spectra below, at, and above the lasing threshold (black, pink, and blue solid line). (b) The integrated intensity as a function of the pumping density.

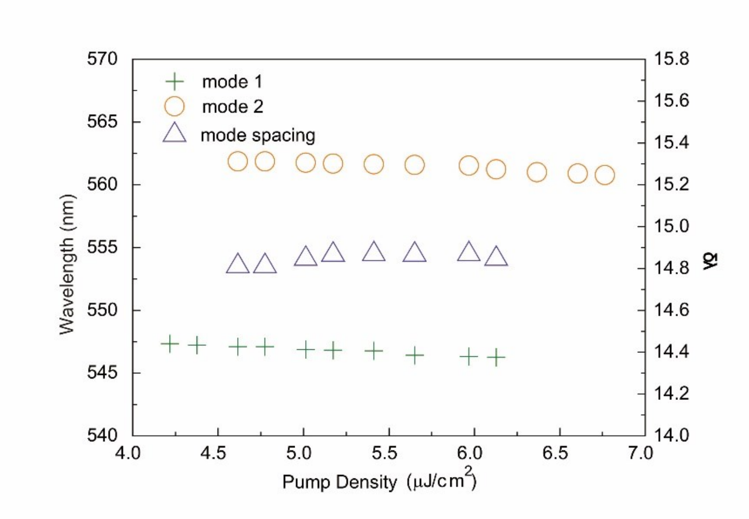

Supplementary Figure 8. The mode spacing as a function of the pump density. Green crosses and orange circles represent the wavelength of mode 1 and mode 2, respectively. Purple triangles represent the mode spacing between these two modes.

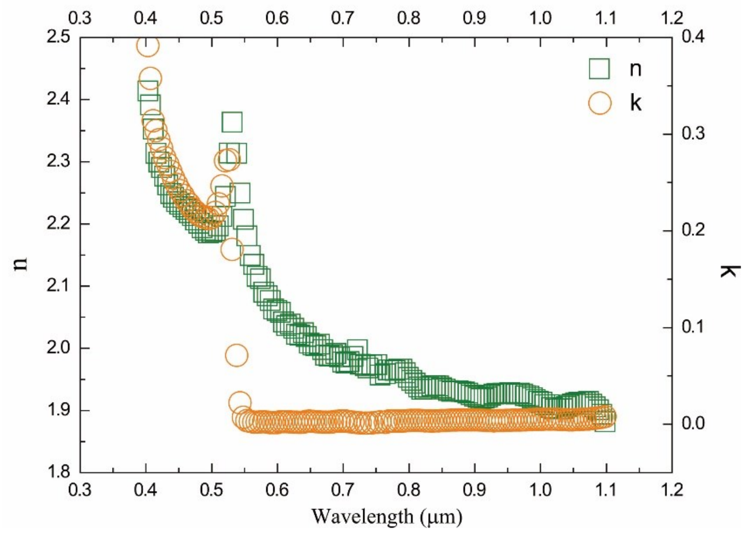

Supplementary Figure 9. Measured refractive index (n, green squares) and light extinction coefficient (k, orange circles) of perovskite microwire.

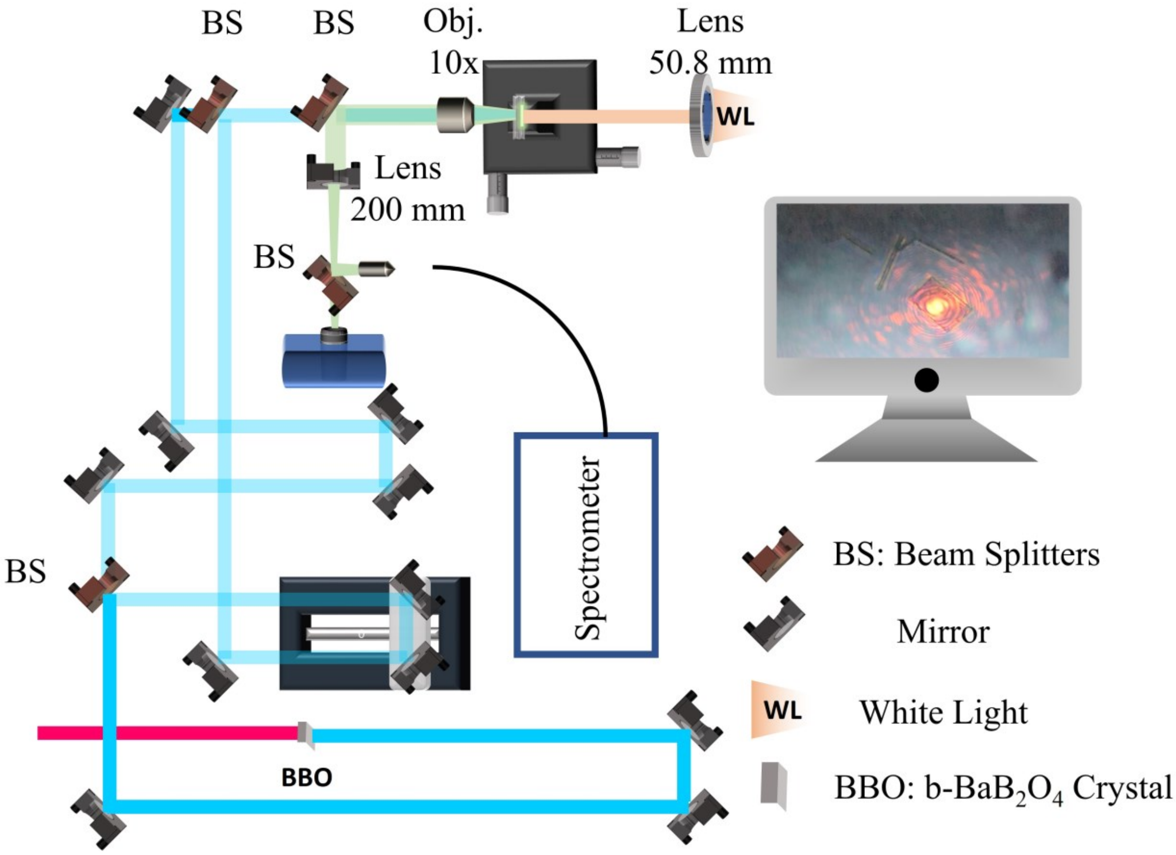

Supplementary Figure 10. Experimental setup measuring the temporal response of mode switching

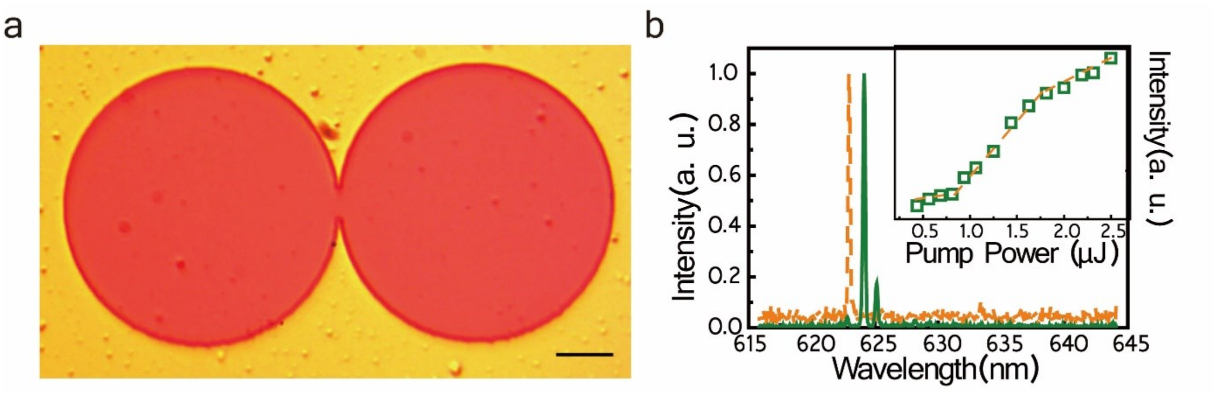

Supplementary Figure 11. Single-mode lasing actions in two coupled microdisks. (a) Top-view microscope image of coupled microdisks. The scale bar is 10 μm. (b) Lasing spectra of coupled microdisks with pump power at 1.25 μJ (dashed line) and 2.50 μJ (solid line). The inset shows the threshold behavior.

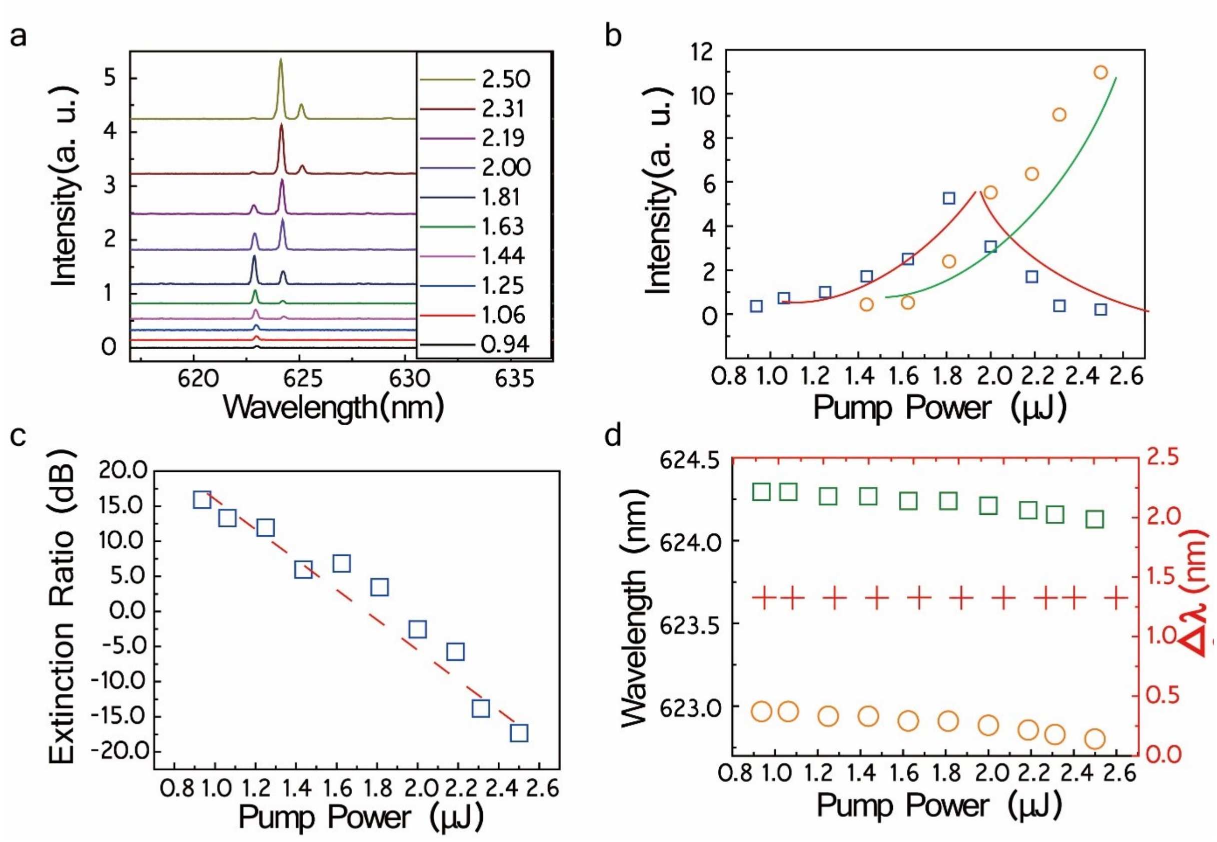

Supplementary Figure 12. Mode switching in coupled microdisks. Laser spectrum (a), peak intensities (b), extinction ratio (c), and lasing wavelengths and mode spacing (d) as a function of the pump power (μJ cm^{-2}).

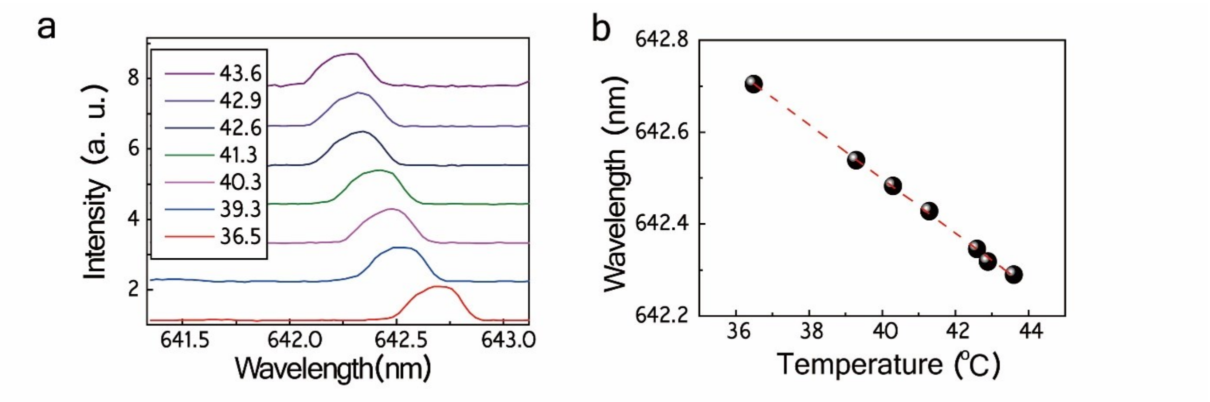

Supplementary Figure 13. The dependence of lase spectrum (a) and peak wavelength (b) on temperature (degree).

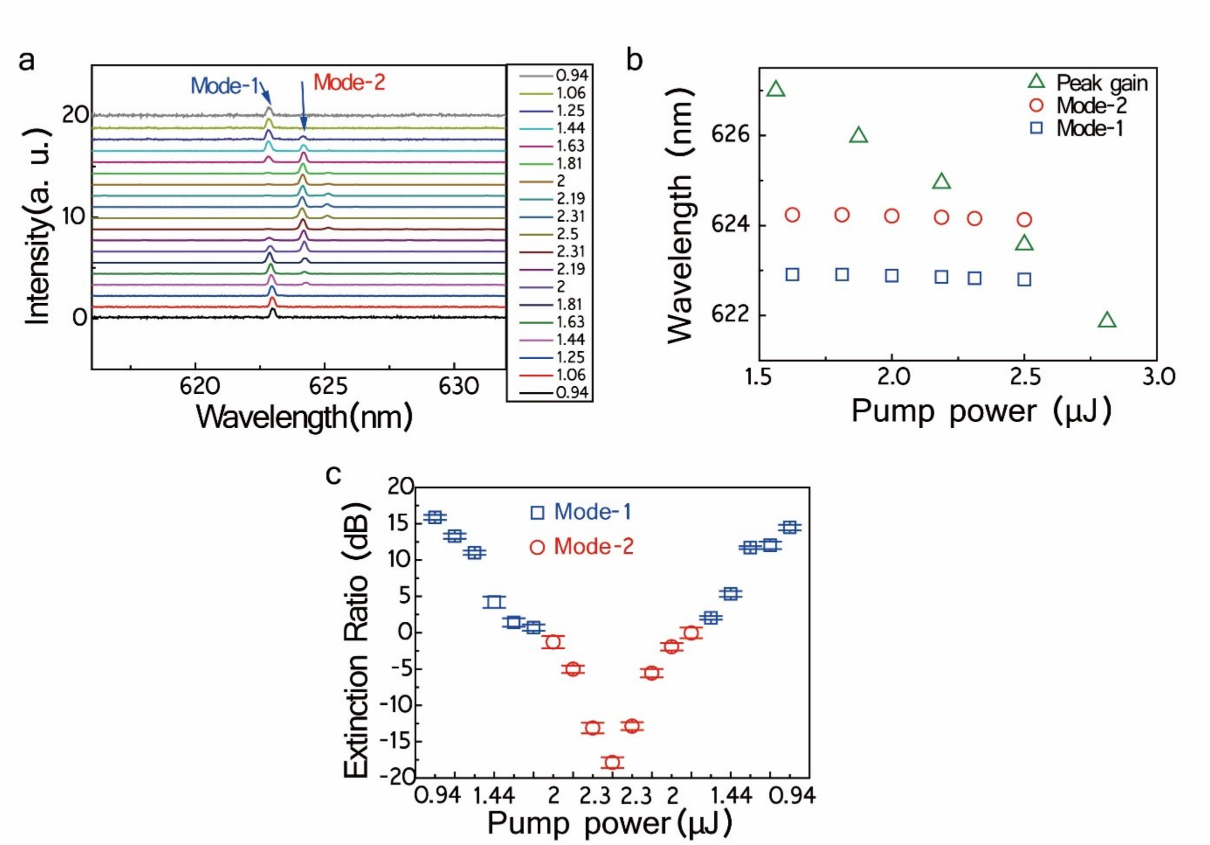

Supplementary Figure 14. Lasing behavior of coupled microdisks under a loop of increasing and decreasing the pump power. (a) Evolution of the spectra. (b) Wavelength shifts of mode-1, mode-2 and peak gain as a function of the pump power (μJ). (c) Extinction ratio along the loop. Blue squares (red circle) represents the dominance of mode-1 (mode-2). The error bar are obtained by statistic the results of 5 times measurements of the same sample.

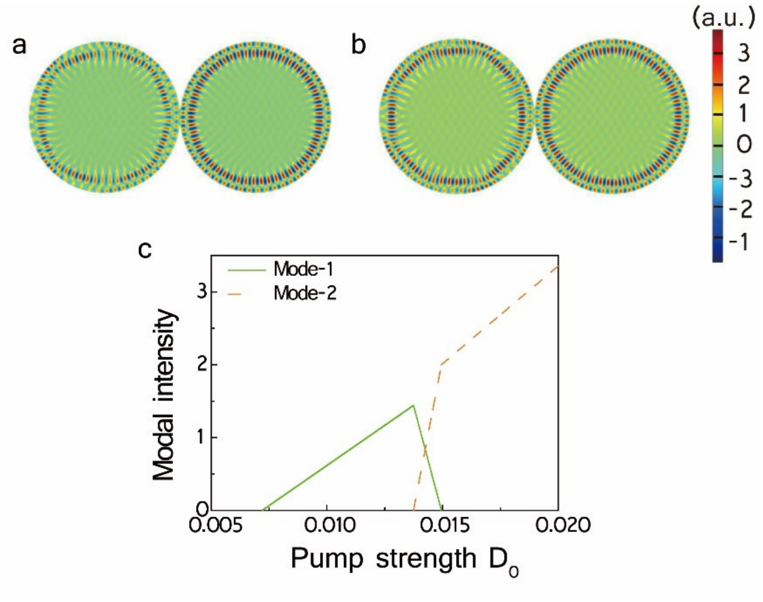

Supplementary Figure 15. Numerical simulation of interaction-induced mode switching in coupled microdisks. (a), (b) Similar field patterns of two modes (mode-1 and mode-2) with close non-interacting thresholds. The azimuthal numbers of mode-1 in the cavities in (a) and mode-2 in the cavities in (b) are m_1=53 and m_2=54, respectively, and their lasing frequencies are k_1R_L=41.7968, k_2R_L =42.4845. (c) Green solid line and orange dashed line show the modal intensities of mode-1 and mode-2, respectively. The parameters used here are: atomic transition frequency k_aR_L=42.1, longitudinal relaxation rate $(\gamma_\perp R_L)/c$=1 and refractive index n=1.56-0.002i.

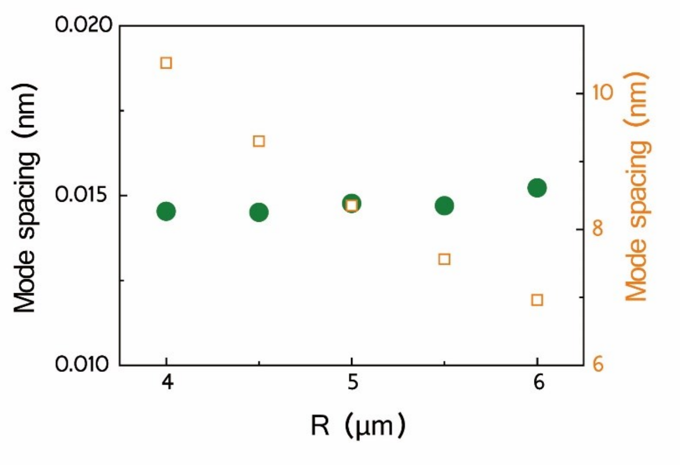

Supplementary Figure 16. The mode spacings as a function of the microcavity radius R. Green circles represent the mode spacing between bonding mode and anti-bonding mode. Yellow squares represent the mode spacing between different longitudinal modes.

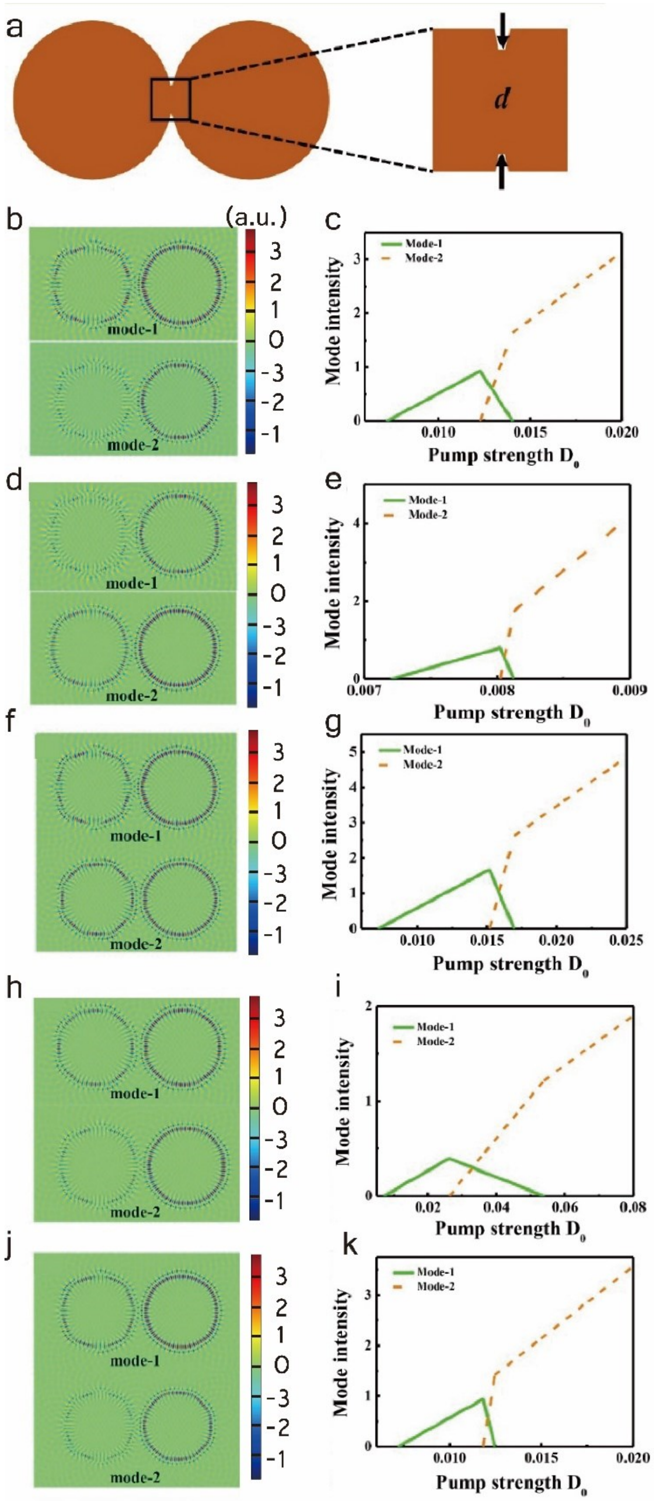

Supplementary Figure 17. (a) The schematic picture of our numerical model. The field patterns (b, d, f, h, j) of two lasing modes and their mode switching (c, e, g, i, k) in coupled microdisks with width 0.91*w* (b, c), 0.95*w* (d, e), 0.97*w* (f, g), 1.05*w* (h, i), and 1.1*w* (j, k). Here *w* is the width in Supplementary Fig. 11.

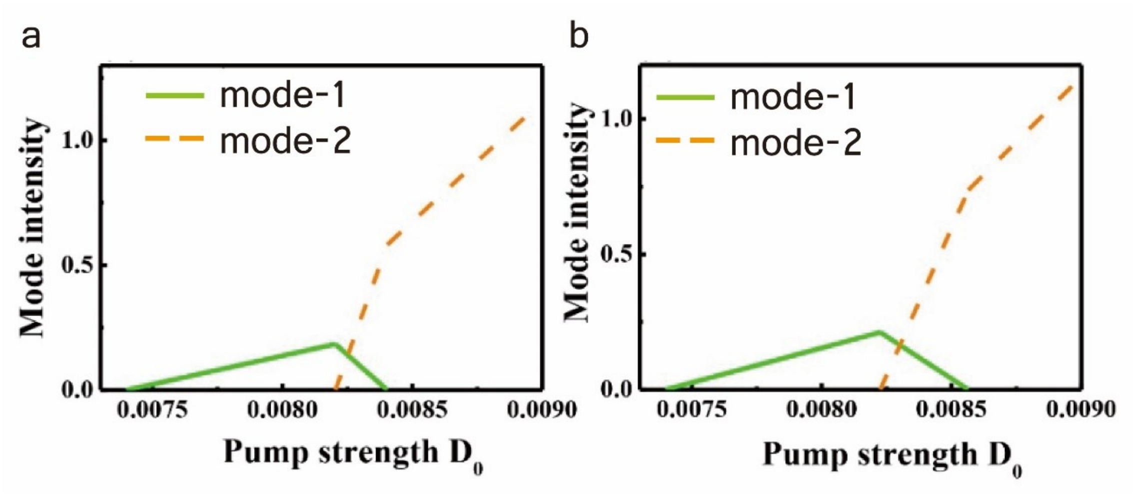

Supplementary Figure 18. Mode switching in coupled microdisks with separation *s*=100 nm (a) and *s*=200 nm (b) between two cavities.

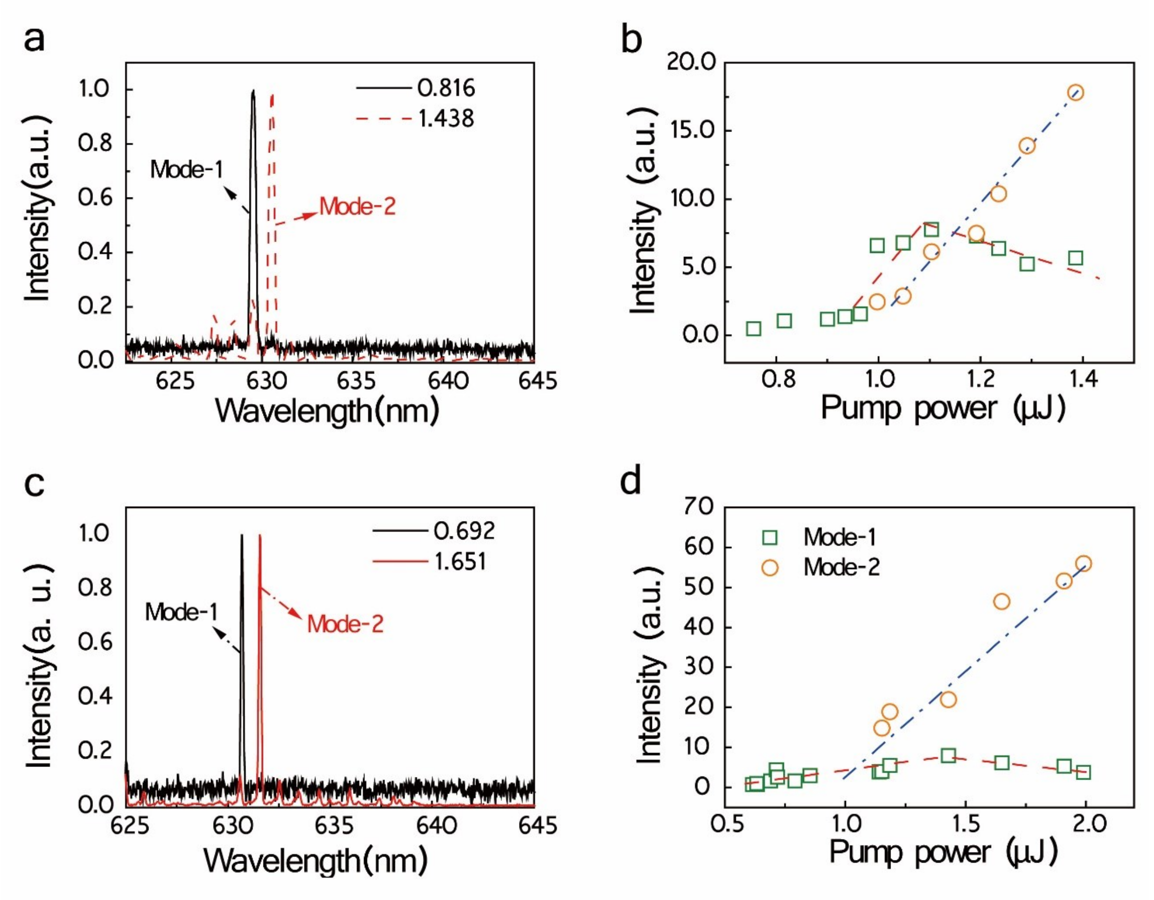

Supplementary Figure 19. Strong modal interaction induced mode switching in different samples around the one in Supplementary Fig. 11. Here (a), (c) show the lasing spectra. And (b), (d) shows the outputs of two lasing modes as a function of pumping power (μJ).

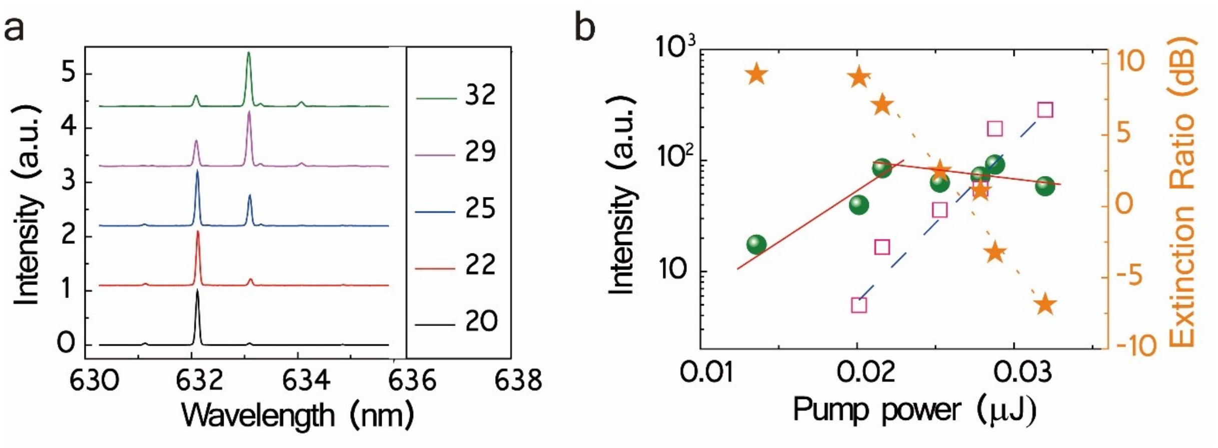

Supplementary Figure 20. Mode switching in another pair of coupled microdisks. (a) The laser spectrum at different pumping power (nJ). (b) The dependence of peak intensities of two lasing modes and their relative difference on the pumping power. Most of the behaviors are similar to Supplementary Figure9 except that single mode laser has been well maintained from threshold P_{th} to $2P_{th}$. The difference between the thresholds in Supplementary Fig. 12 and this figure is mainly induced by the change of optical excitation setup.

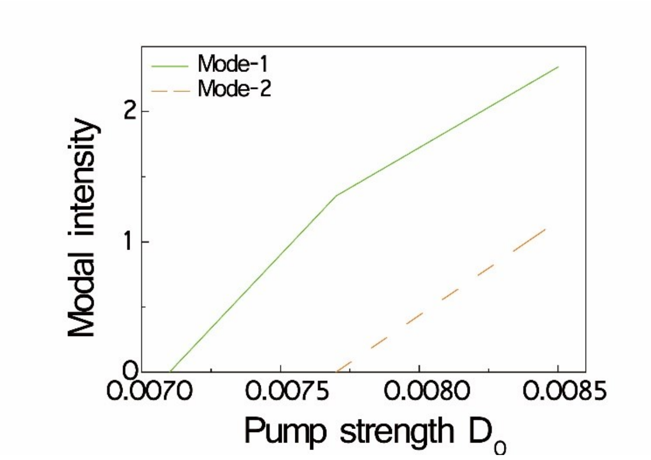

Supplementary Figure 21. Schematic for typical two-mode lasingwith mode competition..

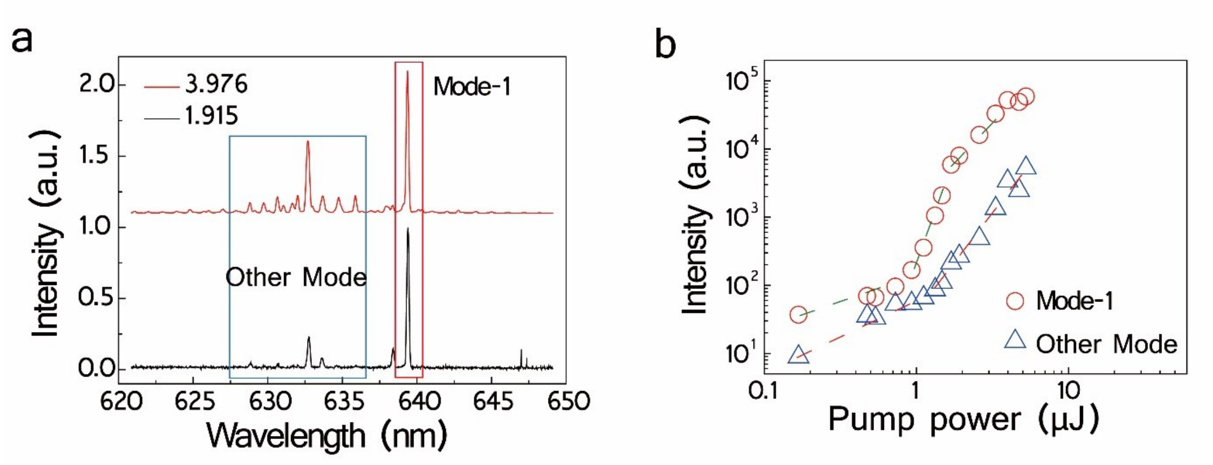

Supplementary Figure 22. The lasing behaviors of coupled microdisks. (a) Lasing spectrum at different pumping power (μJ). (b) The threshold curve of different lasing modes. With the onsets of new lasing modes, the power slopes are slightly reduced, similar to the schematic shown in Supplementary Fig. 21.

Supplementary Note 1: The synthesized $MAPbBr_3$ perovskite microrods

In most cases, synthesized perovskite microwires have a distribution of random sizes based on the fabrication procedure. Here, we made the statistics of the size and thickness of fifty perovskite samples. The distribution of these fifty samples are shown in Supplementary Fig. 1.

Supplementary Note 2: Characterization of the photoluminescence from CH3NH3PbBr3 microwires

In the fluorescence and lasing experiment, the samples were mounted onto a threedimensional translation stage under a home-made microscope and excited by a frequency doubled laser (400 nm, using a b-BaB_2O_4 (BBO) crystal) from a regenerative amplifier (repetition rate1 kHz, pulse width 100 fs, seeded by MaiTai, Spectra Physics). The pump light was focused onto the top surface of the samples through a 40x objective lens and the beam size was adjusted to ∼ 20 μm. The emitted lights were collected by the same objective lens and coupled to a CCD (Princeton Instruments, PIXIS UV enhanced CCD) coupled spectrometer (Acton SpectroPro s2700) via a multimode fiber. The fluorescent microscope images were recorded by a CCD camera behind a longpass filter. The experimental setup is shown in Supplementary Fig. 3.

The optical setup for measuring the absorption of microwires is shown in Supplementary Fig. 4. Basically, the white light is collimated and then focused by a 20× objective lens onto the top surface of the sample, where the diameter of the spot is about 40 μm. The transmitted light is collected by an optical lens and coupled to a spectrometer. The reflected light is collected by the same objective lens and detected by a CCD camera or spectrometer. The CCD camera is used to measure the transmission (T) and reflection (R) from a particular microplate or microwire. The absorption spectrum is calculated by $A = 1 - T - R$. Examples of the absorption spectra of etched/un-etched microwire can be seen in Fig. 1 of the manuscript.

Supplementary Note 3: SALT for modal interactions

To describe our theoretical model, we start with the Maxwell-Bloch (MB) equations in the following form：

$$\nabla^2 E^+ - \frac{1}{c^2}\ddot{E}^+ = \mu_0 \ddot{P}^+, \quad (1)$$

$$\dot{P}^+ = -(-i\omega_a + \gamma_\perp)P^+ + \frac{g^2}{i\hbar}E^+ D, \quad (2)$$

$$\dot{D} = \gamma_\parallel[D_0(\mathbf{r}) - D] - \frac{2}{i\hbar}[E^+(P^+)^* - (E^+)^*]. \quad (3)$$

A 2D cavity with transverse electric polarization is chosen in the system. To simplify the equations, the coupling to negative-frequency is neglected by employing the rotating-wave approximation (RWA). Then under the stationary inversion approximation $\dot{D} \approx 0$, the positive-frequency components of the electric field and polarization of the cavity can be assumed:

$$E^+(\mathbf{r}, t) = \sum_{\mu=1}^{N} \Psi_\mu(\mathbf{r})\, e^{-i\Omega_\mu t},\ P^+(\mathbf{r}, t) = \sum_{\mu=1}^{N} p_\mu(\mathbf{r})\, e^{-i\Omega_\mu t}, \quad (4)$$

where μ=1, 2, … , N represents different lasing modes. The parameters used in Supplementary Equations 1-3 are: $D_0(\mathbf{r})$ is the external pump strength, ω_a is the atomic transition frequency, $\gamma_\parallel$ and $\gamma_\perp$ are the inversion and polarization relaxation rates, $\hbar$ is the reduced Plank constant and g is the transition dipole matrix element between the lasing modes.

We then insert the ansatz Supplementary Eq. (4) into Supplementary Eq. (1)-(3), and the fundamental equations of the SALT are:

$$\left[\nabla^2 + \left(\varepsilon_c(\mathbf{r}) + \frac{\gamma_\perp D(\mathbf{r})}{\omega_\mu - \omega_a + i\gamma_\perp}\right)\omega_\mu^2\right]\Psi_\mu(\mathbf{r}) = 0, \quad (5)$$

$$D(\mathbf{r}) = \frac{D_0(\mathbf{r})}{1+\sum_\mu^N \Gamma_\mu |\Psi_\mu(\mathbf{r})|^2}. \quad (6)$$

Here, $\Gamma_\mu = \gamma_\perp^2/[\gamma_\perp^2 + (\Omega_\mu - \omega_a)^2]$ is the Lorentzian gain curve evaluated at frequency Ω_μ. Ψ and D are measured in their natural units $e_c = \hbar\sqrt{\gamma_\perp\gamma_\parallel}/(2g)$ and $d_c = \hbar\gamma_\perp/4\pi g^2$. Supplementary Equation (5) is a modified Helmholtz equation to describe the behavior of electric field Ψ_μ, and Supplementary Eq. (6) is frequency-dependent and includes infinite-order nonlinear "hole-burning" modal interactions. It is noted that $D_0(\mathbf{r}) = D_0 f(\mathbf{r})$, in which the spatial profile of pump strength D_0 is given by $f(\mathbf{r})$ and normalized by $\int_{cavity} f(\mathbf{r}) d\,\mathbf{r} = A$. For uniform pumping used here in our model, $f(\mathbf{r})$ is equal to 1 inside the cavity and 0 outside the cavity.

At a given pump strength D_0, the lasing mode $\Psi_\mu(\mathbf{r})$ can be expanded as $\Psi_\mu(\mathbf{r}) = \sum_u a_u^\mu \varphi_u(\mathbf{r}, \Omega_\mu)$ at lasing frequency Ω_μ. Unlike conventional eigenvalues in Hermitian system, the constant flux (CF) states $\varphi_u(\mathbf{r}, \Omega_\mu)$ here are biorthogonality and satisfy the following relation:

$$\int_{cavity} d\mathbf{r}\, \varphi_u(\mathbf{r}, \Omega_\mu)\varphi_v(\mathbf{r}, \Omega_\mu) = V\delta_{uv}. \tag{7}$$

To simplify the SALT formula, we assume that the lasing frequency and mode profile are barely changed above the threshold[1], which enables us to employ the single-pole approximation (SPA), $\Psi_\mu(\mathbf{r}) \approx a_u^\mu \varphi_u(\mathbf{r}, \Omega_\mu)$. With the SPA, the derived mode intensities are:

$$\frac{D_0}{D_0^\mu} - 1 = \sum_v \Gamma_v \chi_{uv} I_v. \tag{8}$$

D_0^μ is the lasing threshold without mode interaction. The self-interaction coefficients χ_{11}, χ_{22} and cross-coefficients χ_{12}, χ_{12} are defined as:

$$\chi_{uv} = \frac{1}{A}\left|\int_{cavity} \varphi_u^2(\mathbf{r})\varphi_v^2(\mathbf{r})d(\mathbf{r})\right|. \tag{9}$$

In the calculation, we denote the complex CF frequency $\omega_u = q_u - ik_u$. Then the real-valued lasing frequency of mode μ is given by:

$$\Omega_\mu = \frac{k_u}{k_u+\gamma_\perp}\omega_a + \frac{\gamma_\perp}{k_u+\gamma_\perp}q_u. \tag{10}$$

Below, we refer to $D_{0,\mathrm{int}}^{(\mu)}$ and $D_0^{(\mu)}$ as interacting threshold and noninteracting threshold, respectively. For the first lasing mode, $D_{0,\mathrm{int}}^{(1)} = D_0^{(1)}$. And the non-interacting threshold can be defined as:

$$D_0^{(\mu)} = \frac{2n_0^2}{\Omega_u}\left[1 + \frac{(\omega_a - q_u)^2}{(k_u+\gamma_\perp)^2}\right]. \tag{11}$$

In our theoretical analysis of mode switching, the second mode obviously has a higher threshold, satisfying $D_0^{(2)} > D_0^{(1)}$. The non-interacting power slope, defined by dI_μ/dD_0, can be obtained from Supplementary Equations 8 and 11, i.e., $S_\mu = 1/\Gamma_\mu \chi_{uu} D_0^{(\mu)}$. The power slope S_μ is obviously dependent on the self-coefficients χ_{uu} and threshold $D_0^{(\mu)}$. However, when the second lasing mode is onset at $D_{0,int}^{(2)} = \frac{1-\frac{\chi_{21}}{\chi_{11}}}{\frac{D_0^{(1)}}{D_0^{(2)}} - \frac{\chi_{21}}{\chi_{11}}} D_0^{(1)}$, the mode interaction begins to dominate the lasing behaviors in the system. The

power slope of the initial lasing mode is modified to $\widetilde{S_1} = \frac{\frac{\chi_{22}}{\chi_{12}} - \frac{D_0^{(1)}}{D_0^{(2)}}}{\frac{\chi_{22}}{\chi_{12}} - \frac{\chi_{21}}{\chi_{11}}} S_1$. And before the shutdown of the first lasing mode, the interacting power slope of the second lasing mode is given by $\widetilde{S_2} = \frac{\frac{\chi_{11}}{\chi_{21}} - \frac{D_0^{(2)}}{D_0^{(1)}}}{\frac{\chi_{11}}{\chi_{21}} - \frac{\chi_{12}}{\chi_{22}}} S_2$.

We should note that the interacting threshold $D_{0,\text{int}}^{(2)}$ is increased due to mode interaction, i.e. $D_{0,int}^{(2)} > D_0^{(2)}$.

Actually, a criterion is required to determine whether the interaction-induced mode switch occurs:

$$\frac{\chi_{21}}{\chi_{11}} < \frac{\chi_{22}}{\chi_{12}} < \frac{D_0^{(1)}}{D_0^{(2)}}, \qquad (12)$$

which leads to a negative slope of $\widetilde{S_1}$. Consequently, the initial lasing mode will disappear at

$$D_{0,\text{off}}^{(1)} = \frac{1 - \frac{\chi_{22}}{\chi_{12}}}{\frac{D_0^1}{D_0^2} - \frac{\chi_{22}}{\chi_{12}}} D_0^{(1)} \qquad (13)$$

By applying the SPA-SALT theory, we build a model to describe the lasing behaviors of the two-mode system theoretically, and the results agree well with the experimental observation. When plotting Figs. 4(c) and 4(d), we first extract the self- and cross-interaction coefficients from the field distributions of mode-1 and mode-2 calculated by the finite-element method. These values given in the main text verify that mode switching will take place once the second mode turns on. Next we note that it is difficult to compare the thresholds calculated by SALT and the experimental data directly due to a number of factors, including the surface roughness of the optical cavity. Therefore, to compare the SPA-SALT result with the experimental data in Fig. 2(c), we take $D_0^{(1)} \approx 3\ \mu J/cm^2$ and $D_{0,\text{off}}^{(1)} \approx 5\ \mu J/cm^2$ from the experiment, which tell us that $D_0^{(2)} \approx 3.0004\ \mu\text{J cm}^{-2}$ using Supplementary Eq. (13) and consequently $D_{0,int}^{(2)} \approx 3.7061\ \mu\text{J cm}^{-2}$. The power slope of each mode is then calculated and plotted in Fig. 4(c). Figure 4(d) is plotted using the same parameters by setting $\chi_{11} = \chi_{22} = 0$.

Supplementary Note 4: The analysis of other types of modes for interaction –induced mode switching

In literatures, there are two types of lasers in perovskite micro- & nano-rods. The major type of microlaser in perovskite microrods is the Fabry-Perot lasers such as Supplementary Ref. 2. The other one is the WGM lasers that are generated in the transverse planes of microrods, see Supplementary Ref. 3.

In case of Fabry-Perot lasers, below we will analytically prove that their cross-interaction coefficients χ_{12}, χ_{21} are smaller than their self-interaction coefficients χ_{11}, χ_{22}, which do not meet the requirement of interaction-induced mode switching. In case of Fabry-Perot lasers, below we will analytically prove that their cross-interaction coefficients χ_{12}, χ_{21} are smaller than their self-interaction coefficients χ_{11}, χ_{22}, which do not meet the requirement of interaction-induced mode switching given by Eq. (4) in the main text. To simplify our discussion, we treat them as transverse waves (ψ) described by the one-dimensional (1D) Helmholtz equation:

$$\left[\frac{d^2}{dx^2} + n^2k^2\right]\psi(x) = 0$$

Here x is the axial coordinate of the microrod, n is the refractive index assumed to be uniform inside the microrod between $x = -L/2$ and $L/2$, and k is the free-space wave number. By imposing the outgoing boundary condition at both $x = -L/2$ and $L/2$, i.e.,

$$\psi(x) = \begin{cases} e^{-i\left(k+\frac{L}{2}\right)x} & \left(x < -\frac{L}{2}\right) \\ e^{ik\left(x-\frac{L}{2}\right)} & \left(x > \frac{L}{2}\right) \end{cases}$$

we can solve the modes inside the cavity, which are given by either

$$\psi_-(x) = b\sin(nkx) \quad \text{or} \quad \psi_+(x) = d\cos(nkx). \tag{14}$$

In principle we need to solve for the mode-dependent wave number k and the amplitude b or d simultaneously, but since the latter will be modified using the normalization condition

$$\int_{-\frac{L}{2}}^{\frac{L}{2}} \psi^2(x)dx = L, \tag{15}$$

we eliminate them here and focus on the solutions of k. This is done by employing the continuity boundary condition for the ratio $\frac{\psi'(x)}{\psi(x)}$, which renders the following equation for k:

$$\frac{\psi'_-(x)}{\psi_-(x)} = nk\frac{\cos\left(\frac{nkL}{2}\right)}{\sin(\frac{nkL}{2})} = ik \quad \text{or} \quad \frac{\psi'_+(x)}{\psi_+(x)} = -nk\frac{\sin\left(\frac{nkL}{2}\right)}{\cos\left(\frac{nkL}{2}\right)} = ik$$

or equivalently,

$$\tan(\frac{nkL}{2}) = -in \quad \text{or} \ -\frac{i}{n}$$

The above equation cannot be solved with a real-valued k. Instead, it has discrete solutions in the complex plane, i.e.,

$$k_m = \frac{1}{nL}\left[m\pi - i\ln\frac{n+1}{n-1}\right]$$

where $m = 1,3,5, \dots$ for ψ_- and $2,4,6, \dots$ for ψ_+. These k_m values are known as the (complex) resonances of the modes in a 1D Fabry-Perot cavity, while the corresponding $\psi_\pm(x) \equiv \psi_m(x)$ are called the resonant modes or quasi-bound modes. After the normalization specified by Supplementary Eq. (15), the constant b and d in Supplementary Eq.1(4)are both given by

$$\eta_m = \left[\frac{1}{2} + \frac{i}{(1-n^2)k_m L}\right]^{-\frac{1}{2}}$$

Now it is straightforward to calculate the self- and cross-interaction coefficients, and we show their numerical values in Supplementary Fig. 5. It is clear that the self-interaction coefficients (diagonal bars) are greater than the cross-interaction coefficients (off-diagonal bars), and as a result, the condition for interaction-induced mode switching are not satisfied for Fabry-Perot modes. Even though we have only plotted their values for the 11 modes with the lowest frequency, we show below analytically that this observation holds for any resonances in a 1D Fabry-Perot cavity.

From Supplementary Fig. 5 we find that all self-interaction coefficients are approximately 1.5 while the cross-interaction coefficients are roughly 1. These values can be derived analytically using the approximations

$$k_m \approx \frac{m\pi}{nL}, \quad \eta_m \approx \sqrt{2}$$

For example, for two odd modes

$$\chi_{uv} = \frac{\eta_m^4}{L}\int_{-\frac{L}{2}}^{\frac{L}{2}} \sin(nk_u L)^2 |\sin(nk_v L)^2| dx \approx \frac{4}{L}\int_{-\frac{L}{2}}^{\frac{L}{2}} \sin(u\pi)^2 \sin(v\pi)^2 \, dx = 1 + \frac{\delta_{uv}}{2}$$

and the same results hold. Finally, the cross-interaction coefficients between one even mode v and one odd mode u are given by

$$\chi_{uv} = \frac{\eta_m^4}{L}\int_{-\frac{L}{2}}^{\frac{L}{2}} \sin(nk_u L)^2 |\cos(nk_v L)^2| dx \approx \frac{4}{L}\int_{-\frac{L}{2}}^{\frac{L}{2}} \sin(u\pi)^2 \cos(v\pi)^2\, dx = 1,$$

$$\chi_{vu} = \frac{\eta_m^4}{L}\int_{-\frac{L}{2}}^{\frac{L}{2}} \cos(nk_v L)^2 |\sin(nk_u L)^2| dx \approx \frac{4}{L}\int_{-\frac{L}{2}}^{\frac{L}{2}} \cos(v\pi)^2 \sin(u\pi)^2 dx = 1.$$

These derivations show that indeed interaction-induced mode switching cannot take place between Fabry-Perot modes.

Compared with the Fabry-Perot cavities, the transverse WGMs are much better to realize the mode witching phenomenon. However, there are still several reasons blocking the observation of mode switching frequently. The previous studies on perovskite microrod are intending to synthesize the nanorods. In such systems, the transverse sizes are usually too small to support multiple high Q modes, eliminating the possibility of mode switching between two modes. The most important reason is still the strict criterions. Basically, three conditioned should be fulfilled to realize the mode switching. I) There must be two lasing modes. II) The non-interaction laser thresholds, which is typically determined by the Q factors, must be very close. III) The profiles of two modes must largely overlap to fulfill the equation Eq. (4), which is the criterion for modal interaction induced mode switching. According to the previous experimental results, the transverse sizes of microrods are usually so small that only single-mode WGM lasers have been obtained (see Supplementary Ref. 3). The other transverse modes have much lower Q factors. In addition, the criterion $\frac{\chi_{21}}{\chi_{11}} < \frac{\chi_{22}}{\chi_{12}} < \frac{D_0^{(1)}}{D_0^{(2)}}$ requires that two modes largely overlap one another to ensure strong cross-interactions. This is usually not easy to be fulfilled.

We can simply illustrate this strict requirement with the microrod in Fig. 4 of the manuscript. As shown in Supplementary Fig. 6(a), an additional high Q mode at 533.26 nm can also exist in the transverse plane of microrod. While its mode profile (inset in Supplementary Fig. 6) is still very close the ones in Fig. 4(b), the interaction between mode-1 and mode-3 cannot fulfill the criterion. In other world, this criterion can only be realized with particular modes, if mode-1 and mode-3 are excited in experiment, they will show a regular mode competition instead of mode switching (see Supplementary Fig. 6(b)) although mode switching has been observed between mode-1 and mode-2 in the same

microrod. Without the fine design, even in the large microrods, the mode switching effect is still rare due to the uncontrollability and randomness in chemical synthesis. We need to test hundreds of large perovskite rods to get several useful samples. Therefore, based on the strict requirement of the criterion and the random sizes of as-grown, mode switching is not ubiquitous in the our as-grown perovskite microlasers.

Supplementary Note 5: Lasing action of the perovskite microwire

In this section, we will represent the detail of the lasing action. As shown in Supplementary Fig. 7(a), we give the spectra below and above the lasing threshold, and the integrated intensity as a function of the excitation is summarized in Supplementary Fig. 7(b).

We also discuss the frequency spacing of the two lasing mode. To distinguish this mode switch phenomenon with pump-dependent linear coupling between the two modes, we have carefully measured the wavelength of the two lasing mode and their mode spacing as a function of the pump density. The results are summarized in Supplementary Fig. 8 below. We can see that the mode spacing between these two modes (purple triangles) barely changes with the increase of the pump density, despite the fact that both modes had a minute blue shift at high pump powers, which was a result of band filling effect and has been widely observed in perovskite microlasers.

Supplementary Note 6: Material parameters for numerical simulations of the perovskite microrod

To understand the experimental observations, we performed numerically studies of the experimental sturcuture as mentioned in Methods of the manuscript. The material parameters were taken from the experimental measured refractive index (n) and light extinction coefficient (k) (see Supplementary Fig. 9).

Supplementary Note 7: Experimental setup of two-pulse pump scheme

In the main text, we have demonstrated the temporal response of mode switching. Here we show the measurement setup. The laser source for the 1 kHz two pump pulses experimental setup is based on a Ti: Sapphire oscillator (Mai Tai SP, Spectra-physics) with its output seeding a Ti: Sapphire regenerative amplifier (Spitfire Ace), operating at 1 kHz repetition rate. The regenerative amplifier

provides pulses centered at 800 nm, with 120 fs duration and 5 mJ energy per pulse. In order to measure the laser emission at different time-delays with respect to excitation, two beams were both sent through a controllable delay-stage before focusing it onto the sample to a spot size of ~30 um. The laser emission was detected using a charge-coupled device (Princeton Instrument, PIXIS).

Supplementary Note 8: Experimental observation of interaction-induced mode switching in two coupled polymer microdisks.

In the main text, we have shown that the the mode switching induced by the strong modal interactions in perovskite microrod. In fact, we observed the same phenomenon in another experiment carried out using two coupled microdisks. These coupled microdisks were fabricated by standard photolithography. They had an 8 μm-wide joint region, and their radii were about 40 μm and slightly different (see Supplementary Fig. 11(a)). When both of them were pumped, multiple lasing modes were observed as expected from the large cavity size. When only the left microdisk was pumped, the emission spectrum was dominated by the lasing peak at 622.94 nm above the threshold (around 0.94 μJ; see the inset in Supplementary Fig. 11(b)). The other peaks in the spectrum were suppressed and their intensities were about 10 dB smaller.

An interesting observation was found when the pump power was increased. The initial lasing peak was completely suppressed when the pump power was increased to 2.50 μJ, and another peak at 624.15 nm became the dominant one in the spectrum (see the solid line in Fig. S11(b)). This significant wavelength change was very close to the free spectral range of one microdisk, indicating that the lasing mode was switched to a complete different one instead of between a pair of bonding and anti-bonding modes (see Supplementary Fig. 16).

To better understand this mode switching behavior, we tuned the pump power in smaller steps and recorded the corresponding laser spectra. The results are summarized in Supplementary Fig. 12(a). When increasing the pump power, the intensities of the peaks near 624.15 nm and 622.94 nm crossover, with the former becoming the dominant one above 2.00 μJ. This mode switching process is also plotted in Supplementary Fig. 12(b), where the peak intensities of the two aforementioned modes are plotted as a function of the pump power. The extinction ratio of the spectrum (defined as $10\log I_1/I_2$) is shown

in Supplementary Fig. 12(c), which changed linearly from 20 dB to -20 dB when the pump power was increased from 0.94 μJ to 2.50 μJ. This whole process suggests that the onset of the second lasing mode switched off the initial one.

Similar with the $MAPbBr_3$, we also observed a constant frequency spacing as shown in Supplementary Fig. 12(d). Here the fact that both modes had a minute blue shift at high pump powers was a result of heat accumulation under optical excitation. Once the sample had enough time to release the heat, the lasing frequencies shifted back to their original positions even at higher pump powers. Similar blue shifts were also observed by increasing the evironmental temperature, shown in Supplementary Fig. 13.

Note that the center frequency of the gain curve also experienced a blue shift upon the increase fo the pump power (see Supplementary Fig. 14(b)), and previous studies found that this type of thermal effect can lead to a mode switching behavior, where the new dominant mode at a higher pump power overlaps better spectrally with the shifted gain center[3]. If such a mechanism occurred during the mode switching process we observed, mode 2 shown in Supplementary Fig. 10 should have a shorter wavelength than mode 1. This is exactly opposite to what we found, and hence we can exclude such a thermal effect as the cause of our mode switching behavior.

Again no bistability[4-6] was observed when the pump power was first increased gradually from 0.94 μJ to 2.5 μJ and then reduced back to 0.94 μJ. Due to the power fluctuation of the pump laser, we carried out the intensity integration for five second at each step along the loop, and the averaged spectrum at each pump power is shown in Supplementary Fig. 14(a). The extinction ratio of the two modes along the loop is shown in Supplementary Fig. 14(c), and the absence of bistability is reflected by the good left-right symmetry of this figure. These observations again confirmed that our mode switching behavior was not a result of bistability, and the reversible mode-switching characteristics can be utilized in memory applications.

According to the theoretical analysis in main text, we also numerically studied the lasing actions of these two coupled microdisks. In our calculations, the sizes of two cavities were R_R= 5 μm and R_L = 5.005 μm, and the width of joint region was w = 1 μm. Their refractive index was chosen as 1.56 – 0.002i which includes the intrinsic loss of the dye. Here we take two resonances at k_1R_L = 41.7968 and

k_2R_L = 42.4845 as an example. The corresponding field patterns are depicted in Supplementary Figs. 15 (a) and (b), respectively. Following the SALT calculation, the non-interaction thresholds of the two modes were found to be $D_0^{(1)}$ =0.0072 and $D_0^{(2)}$ =0.0075. The self-interaction coefficients χ_{11}, χ_{22} and the cross-interaction coefficients χ_{21}, χ_{12} were calculated from Eq.(2).

Supplementary Figure 16 illustrates the mode switching behavior of the two lasing modes, and indeed we find that $\chi_{11} = 6.8086\times10^{20}$, $\chi_{12} = 6.2367\times10^{20}$, $\chi_{21} = 6.2367\times10^{20}$ and $\chi_{22} = 5.7555\times10^{20}$ in this example satisfy its requirements given by Eq. (4) in the main text. Therefore, we believe that modal interaction also played a crucial role in mode switching we observed here. We note that the influence of the joint region was also studied, and similar mode switching behaviors were found in a wide range of *w* (see Supplementary Fig. 17).

In addition, we have also numerically studied the mode switching in coupled cavities with different distance between two cavities. Here two samples are selected to investigate the relative amplitudes of the self- and cross-interaction coefficients. The interaction-induced mode switching still can be observed between two longitudinal modes when a gap is introduced between two cavities. We find their values are $\chi_{11} = 6.055 \times 10^{22}$, $\chi_{12} = 2.886 \times 10^{22}$, $\chi_{22} = 1.6470 \times 10^{22}$ and $\chi_{21} = 2.886 \times 10^{22}$ when the separation s=100 nm and $\chi_{11} = 5.4048 \times 10^{22}$, $\chi_{12} = 2.644 \times 10^{22}$, $\chi_{22} = 1.435 \times 10^{22}$ and $\chi_{21} = 2.644 \times 10^{22}$ when *s*=200 nm. The modal intensities are depicted in Supplementary Fig. 18. This result indicates that the mode interaction can occur when the simple requirement given by Supplementary Eq. (12) [and Eq. (4) in the main text] is satisfied.

Based on the numerical calculations, we have studied more samples similar to the one in Supplementary Fig. 11. The strong modal interaction induced mode switching was found to be quite generic (~25 % of checked samples). Some additional results are shown in Supplementary Fig. 19. With the increase of pumping power, we can see that mode-1 was suppressed, whereas mode-2 emerged in the laser spectra (see Supplementary Fig. 19(a)). The corresponding dependences of their intensities on the pumping power is shown in Supplementary Fig. 19(b). Similar to the reports in the main text, the onset of the second lasing modes is associated with the decreasing of the first one. Similar phenomenon also holds true for another samples in Supplementary Figs. 19(c) and 19(d). With the increase of pumping power, the lasing mode switched from 630.7 nm to 631.7 nm. Moreover, we note that the

sample in Supplementary Fig. 20 was fabricated on a different wafer from the one in Supplementary Fig. 12. Thus we know that modal interaction induced mode switching can be easily reproduced and is very generic in coupled microdisks as well.

Mode competition is a general phenomenon in microlasers. Due to the inhomogeneous gain coefficients of lasing materials, some modes reach thresholds easier and lase first. With the increase of pumping power, other lasing modes gradually appear in the laser spectra. And thus the mode competition shall happen, but mode switching will not occur unless Eq. (4) in the main text is satisfied. When the interaction coefficients do no satisfy this relation, the onset of a second lasing mode only slightly reduces the power slope of the first one (see Supplementary Fig. 21), instead of switching it off.

We have done a control experiment to illustrate this conventional result of modal interaction. All the parameters of coupled microdisks are similar to Supplementary Fig. 11 except for slight variations in cavity sizes that are caused by the resolution of photolithography (1 μm for our Mask aligner).

Then the coupled-microdisks are optically excited under the same pumping conditions as Supplementary Fig. 12. Importantly, the pumping configuration is well remained during the whole excitation process. The lasing spectra at different pumping powers have been plotted in Supplementary Fig. 22(a). At lower pumping power, the cavity is dominated by mode-1 and it acts as a single mode laser. When the pumping power is further increased, the other modes increase very quickly and compete with mode-1. The threshold behavior of mode-1 is shown as red circles in Supplementary Fig. 22(b). When the pumping power is above 0.93 μJ, mode-1 becomes lasing and have a power slope around 5.97. Once the other modes appear and start to lase at 1.915 μJ, we can see that the power slope is quickly reduced to around 2.55. This is a very typical mode competition phenomenon in microlasers and quite different from the modal interaction induced mode switching.

Supplementary References

(1) Ge, L. *et al.* Interaction-induced mode switching in steady-state microlasers. *Opt. Express* **24**, 41 (2016)

(2) Zhu, H. et al. Lead halide perovskite nanowire lasers with low lasing thresholds and high quality factors. *Nat. Mater.* **14**, 636-642 (2015).

(3) Wang, K. et al. Formation of single-mode laser in transverse plane of perovskite microwire via micromanipulation. *Opt. Lett.* **41**, 555-558 (2016).

(4) Heumier, T. Mode hopping in semiconductor lasers. (Thesis) Montana State University (1992).

(5) Ishii, S., Nakagawa, A. & Baba, T. Modal characteristics and bistability in twin microdisk photonic molecule lasers. *IEEE J. Sel. Top. Quantum Electron*. **12**, 71 (2006).

(6) Zhukovsky, S., Chigrin, D., Lavrinenko, A. & Kroha, J. Switchable lasing in multimode microcavities. *Phys. Rev. Lett*. **99**, 073902 (2007).

(7) Lv, X. *et al.* Mode characteristics and optical bistability for AlGaInAs/InP microring lasers. *IEEE Photon. Tech. Lett*. **26**, 1703-1706 (2014).
